# Artificial Intelligence Algorithm-Based Lumbar and Spinal MRI for Evaluation of Efficacy of Chinkuei Shin Chewan Decoction on Lumbar Spinal Stenosis

**DOI:** 10.1155/2021/2700452

**Published:** 2021-12-29

**Authors:** Yuefeng Zhu, Tao Wu, Wenhao Wang, Chengchen Cai, Bin Zhu, Weilong Lin, Hao Xu, Qianqian Liang, Yongjun Wang

**Affiliations:** ^1^Longhua Hospital, Shanghai University of Traditional Chinese Medicine, Shanghai 200032, China; ^2^Institute of Spine, Shanghai University of Traditional Chinese Medicine, Shanghai 200032, China; ^3^Department of Orthopedics, Huadong Hospital, Fudan University, Shanghai 200040, China; ^4^Traumatology and Orthopedics of Traditional Chinese Medicine, Huadong Hospital, Fudan University, Shanghai 200040, China

## Abstract

The study aimed to explore the application value of lumbar Magnetic Resonance Imaging (MRI) images processed by artificial intelligence algorithms in evaluating the efficacy of chinkuei shin chewan decoction (a traditional Chinese medicine to nourish the kidney) in the treatment of lumbar spinal stenosis (LSS). Specifically, 110 LSS patients admitted to the hospital were selected as the research subjects. They were randomly divided into the control group (*n* = 55) and experimental group (*n* = 55) according to different treatment methods. The control group was treated with traditional medicine, and the experimental group additionally took chinkuei shin chewan decoction on its basis. Based on the traditional U-net algorithm, a U-net registration algorithm based on artificial intelligence was designed by introducing the information entropy theory, and the algorithm was applied to the lumbar MRI image evaluation of LSS patients. Compared with the traditional U-net algorithm, the artificial intelligence-based U-net registration algorithm had a decreased noise level (*P* < 0.05), the Jaccard (*J*) value (0.84) and the Dice value (0.93) increased significantly versus the traditional algorithm (*J* = 0.63, Dice = 0.81), and the characteristics of the image were more accurate. Before treatment, the Oswestry Disability Index (ODI) scores of the experimental group and the control group were 44.32 ± 6.45 and 43.32 ± 5.45, respectively. After treatment, the ODI scores of the two groups were 10.21 ± 5.05 and 17.09 ± 5.23, respectively. Both showed significant improvement, while the improvement of the experimental group was more obvious than that of the control group (*P* < 0.05). The overall effective rates of the two groups of patients were 96.44% and 82.47%, respectively, and the experimental group was significantly higher than the control group (*P* < 0.05). Under the U-net registration algorithm based on artificial intelligence, the diagnostic accuracy of lumbar MRI in the experimental group was 94.45%, significantly higher than 67.5% before the introduction of the algorithm (*P* < 0.05). In conclusion, chinkuei shin chewan decoction are effective for the treatment of LSS, and lumbar MRI based on the artificial intelligence U-net registration algorithm can evaluate the efficacy of LSS well and is worthy of promotion.

## 1. Introduction

Lumbar spinal stenosis (LSS) is a syndrome of low back pain, lower limb pain, numbness, weakness, and intermittent claudication arising from bony or fibrous stenosis of the spinal canal and compression of the spinal cord, cauda equina, or nerve roots [1, 2]. The incidence of LSS is the second only to lumbar disc herniation among spinal canal diseases [3]. It is mainly divided into developmental stenosis and degenerative stenosis and degenerative spinal stenosis is more common clinically [3]. According to its symptoms, it can be divided into central lumbar spinal stenosis, nerve root canal lumbar spinal stenosis, and mixed lumbar spinal stenosis. According to the cause, it can be divided into primary lumbar spinal stenosis and secondary lumbar spinal stenosis [3]. The main symptoms of LSS are low back pain, lower limb pain, numbness, weakness, intermittent glass line, cauda equina symptoms, and so on [4].

The current imaging diagnostic methods for LSS include X-ray film, computed tomography (CT) examination, and Magnetic Resonance Imaging (MRI) examination [5], among which X-ray examination measures the transverse diameter of the spinal canal and the sagittal diameter of the spinal canal through the lateral view and the transverse diameter less than 18 mm and the sagittal diameter less than 13 mm is considered spinal stenosis [6]. CT imaging is an excellent examination method for lumbar and spinal diseases with a high consistence rate with clinical results. MRI examination can clearly show the spinal canal, extradural fat, dural sac, cerebrospinal fluid, spinal cord, and other structures. It has a higher resolution of soft tissue than CT. In addition to cross-sectional scan, it can also perform sagittal scans. Hence, in the clinical diagnosis of LSS, MRI is the most widely used examination method [7, 8]. In recent years, artificial intelligence algorithms have been widely used in medical imaging [9]. The widely used U-network algorithm is a common medical image segmentation algorithm. It adopts the full convolution neural network and can complete the pixel-level image segmentation, demonstrating good segmentation effects on small datasets. However, there are many problems such as excessive redundancy, slow network training, and incompatible sensitivity field and positioning accuracy [10]. There are many reports on the U-net algorithm of MRI, but there is no report on intelligent optimization algorithm-based MRI examination of LSS. In this study, an optimization algorithm, namely, the artificial intelligence U-net registration algorithm, was incorporated into the MRI of LSS, and it was optimized on the basis of the traditional U-net algorithm.

As a kind of traditional Chinese medicine preparation, chinkuei shin chewan decoction have been confirmed by many reports to have a good effect on LSS [11]. According to research, on the basis of traditional medicine and physiotherapy massage and other treatments, the introduction of chinkuei shin chewan decoction is of great benefit to the improvement of the condition of LSS patients [12]. In this study, the MRI images of the lumbar spine were processed by an artificial intelligence algorithm to evaluate the clinical efficacy of the traditional Chinese medicine chinkuei shin chewan decoction in the treatment of LSS.

In this study, an artificial intelligence-based U-net registration algorithm was innovatively proposed to realize the intelligent processing of lumbar MRI images, and the processed MRI images were used to evaluate the clinical efficacy of chinkuei shin chewan decoction in treating LSS. This study was expected to provide a reference for improving the diagnostic efficiency of clinical lumbar MRI image and a data basis for evaluating the efficacy of chinkuei shin chewan decoction in treating LSS.

## 2. Materials and Methods

### 2.1. Research Subjects

110 LSS patients admitted to the hospital from April 2018 to April 2021 were selected as the research subjects, including 67 males and 43 females. They were aged between 34 and 76 years, and the average age was 42.31 ± 6.25 years. The average duration of all patients was 49.21 ± 11.02 months, including 34 patients with simple intermittent claudication, 52 patients with low back pain and intermittent claudication, 14 patients complicated by lower extremity radiation pain, and 10 patients complicated by sphincter dysfunction. The disease mostly occurred in L2/3, L3/4, L4/5, and L5/S1. Of the 110 patients, there were 5 cases in 3 segments, 48 cases in double segments, and 57 cases in a single segment. All patients underwent the MRI examination of the lumbar spine:

  Inclusion criteria: (i) the patient's clinical manifestations met the diagnostic criteria of LSS; (ii) the patient had no contraindications to the drugs; and (iii) the patient had high compliance with the treatment and there was no abnormality of the mental system.
  Exclusion criteria: (i) patients who had contraindications to the drugs used in the study; (ii) patients with severe systemic diseases; (iii) patients with mental illness; and (iv) patients who had not signed the informed consent. All procedures of this study have been approved by the ethics committee of the hospital, and all subjects included in the study had signed an informed consent form.

### 2.2. Grouping Based on Treatment Methods

In this study, 110 patients were randomly and blindly divided into the control group and experimental group, with 55 people in each group. The two groups of patients were treated with different treatment methods. The control group was treated with traditional medicines (5 mL of Danshen Chuanqiangzine mixed with 250 mL of normal saline for two injections) combined with massage; the experimental group additionally took chinkuei shin chewan decoction on the basis of the control group. One treatment course lasted for 3 weeks, with two courses in total.

### 2.3. Instruments for the MRI Scan

All patients in this study underwent the lumbar MRI examination before operation. The MRI scanner used was Siemens Prisma 3.0 T MRI scanner. The selected spine matrix coil was an 8-channel full spine phased array coil, and routine scanning of the lumbar spine and sacrum 1 vertebral body was performed. The scanning range was from the lower edge of the upper vertebral arch to the upper edge of the lower vertebral arch, including 3 main planes of lateral recess, intervertebral disc, and intervertebral foramen. Scanning sequence and parameters used: (1) FSETWI sequence sagittal position: repetition time (TR) 440 ms, echo time (TE) 9 ms, layer thickness 4 mm, and layer spacing 1 mm; (2) T2WI-IDEAL sequence sagittal position: TR2360 ms, TE87 ms, layer thickness 4 mm, and layer spacing 1 mm; and (3) FSET2WI sequence axial position: TR2780 ms, TE123 ms, layer thickness 3 mm, and layer spacing 0.5 mm. After the scan, the attending physician and MRI diagnostician analyzed the MRI images for a preliminary diagnosis. Observation indicators include pedicle plane, intervertebral foramen plane, ligamentum flavum thickness, intervertebral disc level, and subdisc level.

### 2.4. U-Net Registration Algorithm

**Figure 1:**
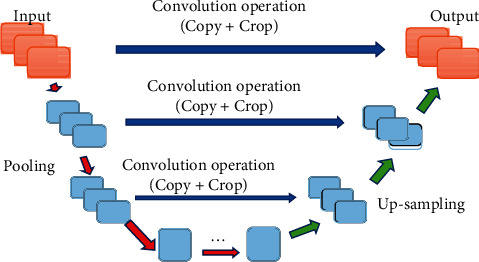
Network model based on U-net.

The design of the lumbar MRI image segmentation algorithm in this study draws on the classic U-net network architecture [13], as shown in Figure 1.

The U-net network architecture includes a contraction link and an expansion link. The link operation rules follow the classic convolutional network architecture. On this basis, a corrective linear unit (ReLU) in each convolution is added to facilitate the trimming when the edge is missing in the convolution operation [14]. During the training of U-net network, the energy function of the softmax is shown as follows:

(1) $$\begin{array}{c} h_{a}\left(z\right)=\frac{\exp\left(s_{a}\left(z\right)\right)}{\left(\sum_{a^{\prime }-1}^{a}\exp\left(s_{a^{\prime }}\left(z\right)\right)\right)}, \end{array}$$

where *s*_*a*_(*z*) means that the pixel position, *z*∈Ω and Ω⊂*Z*_2_, *a* means the number of segmentation classes, and *h*_*a*_(*z*) means the maximum function value.

In this study, the objective function based on the Dice coefficient replaces the cross-entropy loss function in the U-net network training process. Then, based on the characteristics of the five vertebrae of the lumbar spine in the LSS patient [15], the lumbar spine MRI images of LSS patients are further registered. First, the reference image *A* and the floating image *B* are set, and the relevant expressions for the registration minimization are shown as follows:

(2) $$\begin{array}{c} \hat{G}=\arg\underset{G}{\min}C\left(G;A,B\right), \end{array}$$

(3) $$\begin{array}{c} C\left(G;A,B\right)=W\left(G_{\mu };A,B\right)+\lambda Q\left(G_{\mu }\right), \end{array}$$

(4) $$\begin{array}{c} \hat{\mu }=\arg\underset{\mu }{\min}C\left(\mu ;A,B\right), \end{array}$$

where *G* represents the transformation model, *λ* represents the weighting coefficient of the rule item, *μ* represents the parameter vector of the transformation coefficient, and *G*_*μ*_ represents the transformation model containing parameter *μ*.

Next, the lumbar MRI image of the LSS patient is transformed into the basic grid of the control points of the target area, and the equation is as follows:

(5) $$\begin{array}{c} G_{l}\left(x,y,z\right)=\sum_{l=0}^{3}\sum_{a=0}^{3}\sum_{b=0}^{3}R_{l}\left(n_{l}\right)R_{a}\left(n_{a}\right)R_{b}\left(n_{b}\right)\phi_{d+l,e+a,f+b}, \end{array}$$

where *R*_*l*_ represents the *l* basis function, *d*, *e*, *f* represent the control point number on the basic grid, (*x*, *y*, *z*) are the coordinates of any point, and the *n* basis function is in the range of [0, 1). Then, basis functions from *R*_0_ to *R*_3_ can be expressed as follows:

(6) $$\begin{array}{c} R_{0}\left(n\right)=\frac{{\left(1-n\right)}^{3}}{6},\\ R_{1}\left(n\right)=\frac{\left(3n^{3}-6n^{2}+4\right)}{6},\\ R_{2}\left(n\right)=\frac{\left(-3n^{3}+3n^{2}+3n+1\right)}{6},\\ R_{3}\left(n\right)=\frac{n^{3}}{6}. \end{array}$$

**Figure 2:**
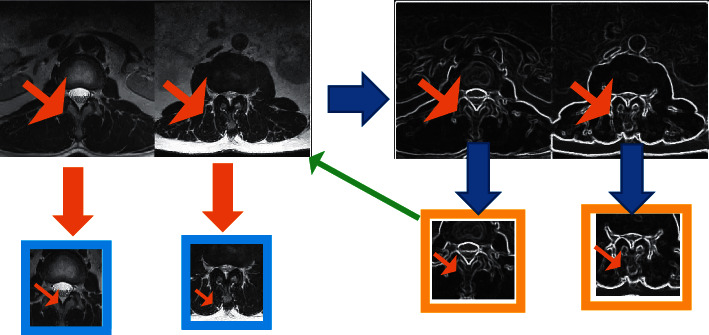
Mutual information algorithm processing flowchart based on U-net image registration.

At the same time, a mutual information algorithm is proposed based on the theory of information entropy.  Figure 2 is a flowchart of the lumbar MRI image processing, and the mutual information is calculated as equation (8).

(7) $$\begin{array}{c} \text{MI}\left(A,B\right)=K\left(A\right)+K\left(B\right)-K\left(A,B\right), \end{array}$$

(8) $$\begin{array}{c} \text{MI}\left(A,B\right)=\sum_{a\in A}\sum_{b\in B}p\left(a,b\right)\log\left(\frac{p\left(a,b\right)}{p\left(a\right)p\left(b\right)}\right), \end{array}$$

where *A* and *B* are the two given images, *K*(*A*) and *K*(*B*) are the edge entropy of the two images, respectively, *K*(*A*, *B*) is the joint entropy of the two images, *p*(*A*, *B*) is the joint probability function of *A* and *B*, and *p*(*a*) and *p*(*b*) are the marginal probability distribution functions of *A* and *B*, respectively. On this basis, the distortion energy penalty term [16] is added, and a similar cost function can be obtained, expressed as follows:

(9) $$\begin{array}{c} C\left(\mu \right)=\alpha_{1}F\left(\mu \right)+\alpha_{2}V\left(\mu \right), \end{array}$$

where *α*_1_, *α*_2_ are self-defined weighting constants, *F* is the traditional similarity measurement function, *C*(*μ*) is the upgraded version of the similarity measurement function, and *V* is the distortion energy penalty item. The second derivative of the energy penalty term is expressed as follows:

(10) $$\begin{array}{c} V\left(\mu \right)=\int_{u}\left[{\left(\frac{\partial^{2}G}{\partial x^{2}}\right)}^{2}+{\left(\frac{\partial^{2}G}{\partial y^{2}}\right)}^{2}+{\left(\frac{\partial^{2}G}{\partial z^{2}}\right)}^{2}+2{\left(\frac{\partial^{2}G}{\partial x\partial y}\right)}^{2}+2{\left(\frac{\partial^{2}G}{\partial x\partial z}\right)}^{2}+2{\left(\frac{\partial^{2}G}{\partial z\partial y}\right)}^{2}\right], \end{array}$$

where *u* represents the spatial image domain. Then, equation (10) is discretized to obtain

(11) $$\begin{array}{c} V\left(\mu \right)=\frac{1}{M_{u}}\sum_{X\in u}\Phi_{G}\left(X\right), \end{array}$$

where *M* represents the number of spatial image points and Φ is the sum of the squares of the second derivative of point *x* under the transformation *G*.

### 2.5. Quality Evaluation of Lumbar MRI Images Processed by U-Net Registration Algorithm

Because there is no publicly available gold standard database for the lumbar spine images that can be used to evaluate the registration accuracy, in the study, we adopt the coincidence degree between anatomical structure and tissue and target registration error (TRE) to evaluate the accuracy of the algorithm. The Dice (*D*) coefficient and the voxelwise Jaccard (*J*) index value were used to calculate the coincidence degree of the vertebrae and blood vessels. The calculation equations were as follows: the accuracy took the mean value of the target registration error corresponding to the mark point, expressed as follows:

(12) $$\begin{array}{c} \text{Dice}=\frac{2|Q\cap W|}{|Q|\cup |W|},\\ J=\frac{|Q\cap W|}{|Q\cup W|},\\ m\text{ TRE}=\frac{1}{l}\overset{l}{\underset{j}{\sum }}\left\|G\left(x_{i}\right)-x_{i}^{\prime }\right\|. \end{array}$$

### 2.6. Efficacy Evaluation of Chinkuei Shin Chewan Decoction and Diagnostic Value of Artificial Intelligence-Based Lumbar Spine MRI

The lumbar MRI was mainly used to evaluate the efficacy. In addition, the two groups of patients were compared for the Oswestry Disability Index (ODI) scores (basic daily activities, participation in basic social activities, Pain index, etc.) to comprehensively evaluate the therapeutic effect of chinkuei shin chewan decoction on patients with LSS.

### 2.7. Statistical Methods

The data were processed by SPSS 19.0 statistical software. The measurement data were expressed as mean ± standard deviation $\left(\bar{x}\pm s\right)$. The comparison of the means between groups adopted *t*-test. The count data were expressed by the percentage (%), and the *χ*2 test was used. *P* < 0.05 indicates that the difference was statistically significant.

## 3. Results

### 3.1. Comparison of Basic Data of the Two Groups of Patients

**Figure 3:**
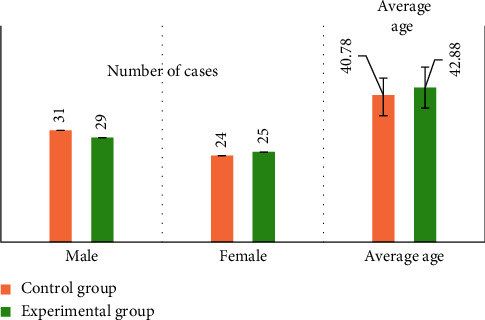
The distribution of gender and the average age of the two groups of patients before treatment.

**Figure 4:**
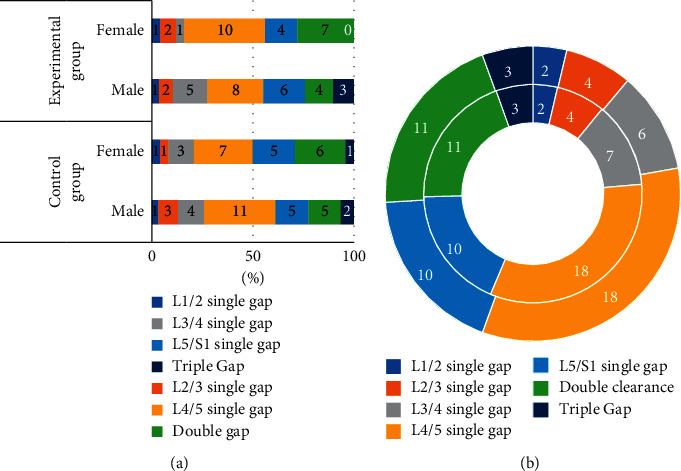
The distribution of the diagnosis results of the lumbar MRI lesions of the two groups of patients before treatment. (a) The gender distribution of the two groups of patients. (b) The distribution of the lumbar MRI lesions of the two groups of patients, where the inner ring represents the experimental group and the outer ring represents the control group.

**Figure 5:**
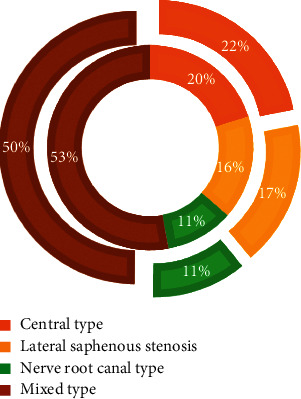
The distribution of the diagnosis results of the lumbar spine MRI lesion types before treatment in the two groups of patients. (Note: the inner ring represents the experimental group and the outer ring represents the control group.)

The basic information of the two groups of patients was compared, as shown in Figures 3–5.  Figure 3 shows the gender distribution and average age distribution of the two groups of patients before treatment, Figure 4 shows the distribution of the diagnosis results of the lumbar spine MRI lesions of the two groups before treatment, and Figure 5 shows the diagnostic results of the types of lumbar MRI lesions of the two groups of patients before treatment. According to Figure 3, in the control group, there were 31 males and 24 females; in the experimental group, there were 29 males and 25 females. In the control group, the average age was 40.78 ± 4.73 years, and in the experimental group, the average age was 42.88 ± 5.69 years. There was no significant difference between the two groups in age distribution (*P* > 0.05). As shown in Figure 4, in the experimental group, there were 2, 4, 6, 18, 10, 11, and 3 patients with LSS lesions in the L1/2 single space, L2/3 single space, L3/4 single space, L4/5 single space, L5/S1 single space, double space, and triple space, while in the control group, the corresponding number was 2, 4, 7, 18, 10, 11, and 3, and the difference was not statistically significant (*P* > 0.05). As shown in Figure 5, in the experimental group, there were 21.8%, 17.2%, 11.2%, and 49.8% patients with central stenosis, lateral recess stenosis, neural tube stenosis, and mixed stenosis, respectively; in the control group, the corresponding number was 20.1%, 16.4%, 10.6%, and 52.9%, and the difference between the two groups was not statistically significant (*P* > 0.05).

### 3.2. Quality Evaluation of U-Net Registration Algorithm-Based Lumbar MRI Images

**Figure 6:**
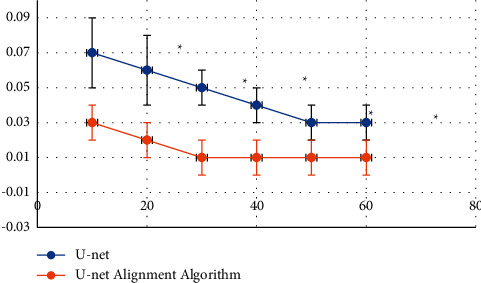
Noise distribution of different algorithms under different current conditions.

**Figure 7:**
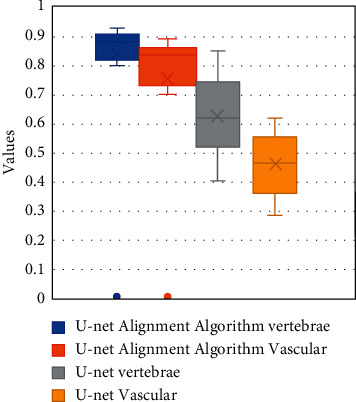
*J* value distribution of vertebrae and blood vessel images under different algorithms.

**Figure 8:**
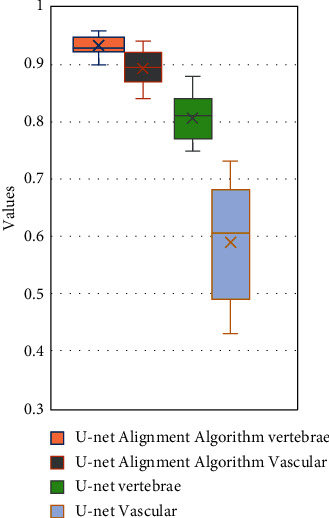
Dice value distribution of vertebrae and blood vessel images under different algorithms.

The traditional U-net algorithm and the artificial intelligence-based U-net registration algorithm were compared for the noise, Dice value, and *J* value under different currents, as shown in Figures 6–8.  Figure 6 shows the noise distribution of different algorithms under different current conditions.  Figure 7 shows the *J* value distribution of vertebrae and blood vessel images under different algorithms.  Figure 8 shows the distribution of Dice values of vertebrae and blood vessel images under different algorithms.

As shown in Figure 6, the noise values of the image processed by the traditional U-net algorithm are 0.07 ± 0.02, 0.06 ± 0.02, 0.05 ± 0.01, 0.04 ± 0.01, 0.03 ± 0.01, and 0.03 ± 0.01 under the current intensity of 10, 20, 30, 40, 50, and 60 mAs, which were significantly higher than 0.03 ± 0.01, 0.02 ± 0.01, 0.01 ± 0.01, 0.01 ± 0.01, 0.01 ± 0.01, 0.01 ± 0.01 under the artificial intelligence-based U-net registration algorithm, and the difference was statistically significant (*P* < 0.05). As shown in Figures 7 and 8, the box plot of the vertebra *J* value under the artificial intelligence-based U-net registration algorithm was short, and the registration result of each data had a small difference, but the blood vessel *J* value was long. Compared with the *J* value of the traditional U-net algorithm, the *J* value of the lumbar MRI images processed by the artificial intelligence-based U-net registration algorithm was significantly increased. The same went for the Dice value. Compared with the Dice value of the traditional U-net algorithm, the Dice value of the lumbar MRI image processed by the artificial intelligence-based U-net registration algorithm was significantly increased.

### 3.3. Comparison of MRI Image Features before and after Processing by Artificial Intelligence-Based U-Net Registration Algorithm

**Figure 9:**
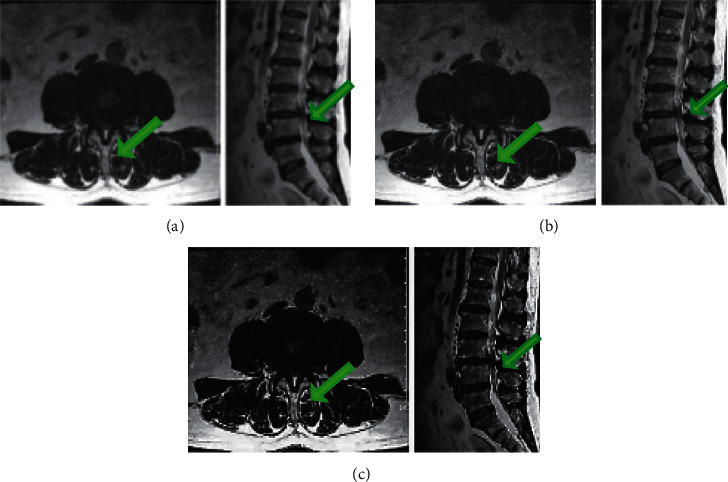
MRI images of the lumbar spine of LSS patients processed by different algorithms. (a–c) The conventional lumbar spine MRI image, the lumbar spine MRI image processed by the U-net algorithm, and the lumbar spine MRI image processed by the artificial intelligence-based U-net registration algorithm; the green arrow points to the lesion.

The lumbar MRI images before and after processing by the traditional U-net algorithm and the artificial intelligence-based U-net registration algorithm were compared, and the results are shown in Figure 9. Figure 9 shows that for the same patient, compared with the original lumbar MRI images, the vertebral imaging effect and clarity after processing by the two algorithms were significantly improved, and the positioning of focus of vertebral stenosis was more accurate. The effects of the artificial intelligence-based U-net registration algorithm were further improved compared to the traditional U-net algorithm.

**Figure 10:**
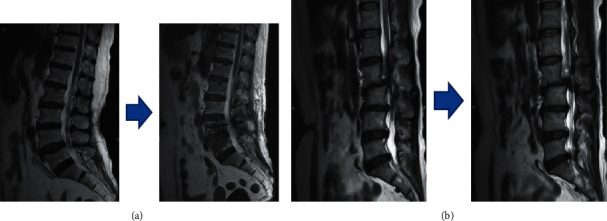
Comparison of MRI images of the lumbar spine before and after treatment between the two groups. (a) MRI images of the lumbar spine before and after treatment in the experimental group. (b) MRI images of the lumbar spine before and after the treatment in the control group.

Figure 10 shows that the posterior edge hyperplasia, osteophytes, articular process hyperplasia, and articular process hyperplasia of the lumbar spine stenosis of the patients in the experimental group were significantly improved, and the anteroposterior and transverse diameters were restored to the normal. Although the lumbar spine stenosis of the patient in the control group was also relieved to a certain extent, the recovery effect was not as good as that of the experimental group.

### 3.4. Evaluation of the Efficacy of Chinkuei Shin Chewan Decoction in the Treatment of LSS

**Figure 11:**
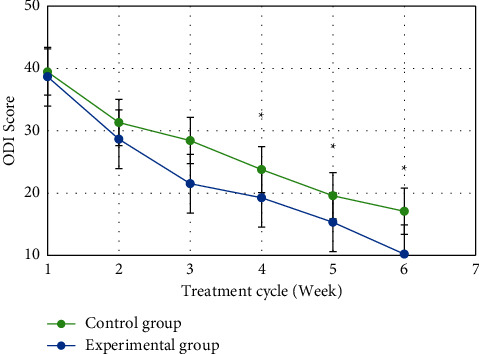
Comparison of ODI scores between the two groups of patients at each stage of treatment. (Note: ^*∗*^ indicates that the difference was significant compared with the control group (*P* < 0.05).)

Figure 11 shows the ODI scores of the two groups of patients at different treatment stages. It was noted that the ODI scores of patients in the control group at 0, 1, 2, 3, 4, 5, and 6 weeks after treatment were 43.32 ± 5.45, 39.45 ± 5.67, 31.33 ± 6.61, 28.43 ± 4.88, 23.78 ± 4.97, 19.58 ± 5.32, and 17.09 ± 5.23, respectively, while the ODI scores of patients in the experimental group were 44.32 ± 6.45, 38.67 ± 5.22, 28.65 ± 5.18, 21.52 ± 4.89, 19.25 ± 4.96, 15.32 ± 4.65, and 10.21 ± 5.05. The ODI scores of patients in the experimental group were significantly lower than those in the control group at 4 weeks, 5 weeks, and 6 weeks after treatment (*P* < 0.05).

**Figure 12:**
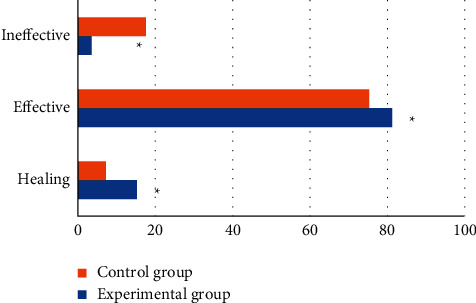
Comparison of curative effects between the two groups of patients. (Note: ^*∗*^ indicates a statistical difference compared with the control group, *P* < 0.05.)

Next, the treatment effect of the two groups of patients was evaluated. As shown in Figure 12, the number of patients cured in the experimental group accounted for 15.21%, the number of effective cases accounted for 81.23%, and the number of ineffective cases accounted for 3.56%, while in the control group, the cured patients accounted for 7.21%, the effective cases accounted for 75.26%, and the ineffective cases accounted for 17.53%. The effective rate of treatment in the experimental group was significantly higher than that in the control group, and the difference was statistically significant (*P* < 0.05).

### 3.5. Application Value of the Algorithm in Evaluating Curative Effects

**Figure 13:**
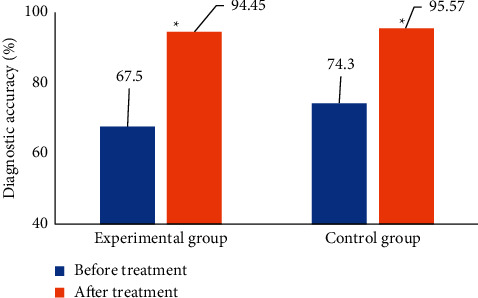
Comparison of SAR scores between the two groups of patients before and after treatment. (^*∗*^ indicates that there was a statistical difference compared with before the introduction of the algorithm, *P* < 0.05.)

As shown in Figure 13, the diagnostic accuracy of the lumbar MRI image was 67.5% in the experimental group and 74.3% in the control group before the image was processed by the U-net registration algorithm based on artificial intelligence. After the introduction of the U-net registration algorithm based on artificial intelligence, the diagnostic accuracy of the experimental group and control group was improved to 94.45% and 95.57%, respectively. Therefore, after the introduction of the U-net registration algorithm based on artificial intelligence, the diagnostic accuracy of both groups was significantly improved, and the difference was statistically significant (*P* < 0.05). There was no significant difference in diagnostic accuracy between the two groups before and after the introduction of the algorithm (*P* > 0.05).

## 4. Discussion

LSS refers to the stenosis of the lumbar spinal canal or intervertebral foramina caused by congenital or acquired factors, which can cause compression of the lumbar spine nerve tissue, blood circulation disorders, pain in the buttocks or lower limbs, and neurogenic claudication, often accompanied by symptoms of waist and leg pain [17, 18]. In clinical treatment, traditional Chinese medicine is extensively used [19]. Traditional massage therapy combined with traditional Chinese medicine has been confirmed by many studies to have a good effect on LSS. The research of Oka et al. confirmed that, according to the ZCQ body function score, the therapeutic effect of acupuncture was better than that of physical exercise, and the satisfaction score of acupuncture was significantly higher than that of drug therapy [20], consistent with the results of Qin et al. [7] and Hadianfard et al. [21].

In the study, it was found that compared with the traditional U-net algorithm, the artificial intelligence-based U-net registration algorithm had a decreased level of noise in processing images (*P* < 0.05), while its *J* value and Dice value increased significantly, and the characteristics of the lesion site of LSS were more accurate. Before treatment, the ODI scores of the experimental group and the control group were 44.32 ± 6.45 and 43.32 ± 5.45, respectively. After treatment, the ODI scores of the two groups were 10.21 ± 5.05, 17.09 ± 5.23, and both showed significant improvement, but the improvement in the experimental group was more obvious (*P* < 0.05). The overall effective rates of the two groups of patients were 96.44% and 82.47%, respectively, and the experimental group was significantly better than the control group (*P* < 0.05). Under the artificial intelligence-based U-net registration algorithm, the diagnostic accuracy of lumbar spine MRI for the two groups increased significantly compared to before the introduction of the algorithm (*P* < 0.05). This suggested that the chinkuei shin chewan decoction made of Rehmannia, Poria, Cornus, Chinese Yam, Cinnamon Sticks, Paeonol, Alisma, and Aconite can nourish the kidney, balance yin and yang, dredge the meridians, and invigorate qi and blood [22, 23], thus effectively alleviating the symptoms of LSS patients. The artificial intelligence-based U-net registration algorithm can reduce image noise, enhance the imaging effect of the vertebrae and blood vessels of LSS patients, and improve the accuracy of the evaluation of LSS treatment effects. It has a good clinical application prospect, but its performance needs to be further optimized.

Artificial intelligence-based U-net registration algorithm can reduce the noise in the lumbar MRI image, enhance the imaging effect, and elevate the accuracy of LSS efficacy evaluation. Lee et al. [24] also improved the U-net algorithm and proposed a patchwise U-net architecture for automatic segmentation of brain structures in structural MRI. The nonoverlapping patchy U-net retained more local information and significantly improved the Dice similarity coefficient versus the traditional U-net, which indicates that the improved U-net has a good application prospect in the field of clinical MRI image diagnosis, but its algorithm performance needs to be further optimized.

## 5. Conclusion

In this study, the artificial intelligence-based U-net registration algorithm was used to process lumbar MRI images of LSS patients to evaluate the efficacy of chinkuei shin chewan decoction in the treatment of LSS. The results showed that the lumbar MRI based on the artificial intelligence U-net registration algorithm demonstrated a good diagnostic value in evaluating the efficacy of LSS, and the therapeutic effect of chinkuei shin chewan decoction on LSS was further verified. However, some limitations in the study should be noted. The sample size is small, which will reduce the power of the study. In the follow-up, an expanded sample size is necessary to strengthen the findings of the study. In conclusion, the artificial intelligence-based U-net registration algorithm demonstrates good application value in evaluating the efficacy of chinkuei shin chewan decoction in the treatment of LSS, which provides a reference for the treatment of LSS patients.

## Data Availability

The data used to support the findings of this study are available from the corresponding author upon request.
